# A comprehensive guide to selecting suitable wavelet decomposition level and functions in discrete wavelet transform for fault detection in distribution networks

**DOI:** 10.1038/s41598-024-82025-2

**Published:** 2025-01-07

**Authors:** Esraa M. Shalby, Almoataz Y. Abdelaziz, Eman S. Ahmed, Basem Abd-Elhamed Rashad

**Affiliations:** 1 Faculty of Engineering, Ain Shams University, Cairo, 11517 Egypt; 2 Department of Electrical Power and Machines Engineering, The Higher Institute of Engineering at El- Shorouk City, El-Shorouk Academy, Cairo, 11837 Egypt; 3 Faculty of Engineering and Technology, Future University in Egypt, Cairo, 11835 Egypt; 4 Department of Electrical Engineering, Faculty of Engineering, Kafrelsheikh University, Kafrelsheikh, Egypt

**Keywords:** Decomposition level selection, Discrete wavelet transform (DWT), Distribution networks, Fault detection, Feature extraction, MATLAB classifier learner, MATLAB wavelets toolbox, Mother wavelets selection, Support vector machine, Electrical and electronic engineering, Scientific data

## Abstract

The paper presents a comprehensive analysis of the IEEE-16 bus system under different operating conditions. It discusses the selection of suitable decomposition level and wavelet function for analyzing non-stationary signals to enhance power distribution network fault detection. MATLAB/Simulink is used to simulate the system, and transient fault current signals are processed with the MATLAB Wavelet Toolbox. The optimal decomposition level is determined by energy concentration, with the highest energy found in scales D9 (b4), D8 (b5), and D7 (b6), and D8 having the most concentration. Using MATLAB classifier learner, the article evaluates seven common mother wavelets with 53 wavelet functions, and sym3 is found to be the most efficient wavelet function in terms of training time, prediction speed, and accuracy of SVM classifiers. All fault types both symmetrical/unsymmetrical types, and various normal transient conditions such as load/capacitor/DG switching are detected/discriminated with nearly 100% accuracy at the midpoint of line 6–7 with various fault conditions, inception angles (0, 30, 45, 60, 90 and 120°) and a fault resistance of (5,10, 15, and 20 ohms). Additionally, 9 MW wind Farm is integrated at busbar 10, and various fault scenarios are simulated to assess system performance with 100% Accuracy.

A consistent provision of electricity stands as a cornerstone in today’s society. Distribution networks are essential to the effective and dependable delivery of electricity to final consumers. A critical challenge in the protection of distribution networks is the accurate identification and isolation of faults to prevent system disruptions. This study specifically targets distribution networks because they are more prone to faults due to their complex topology, high penetration of distributed energy resources, and frequent exposure to external disturbances^1,2^. Moreover, fault detection in distribution networks has been an active area of research in recent years as reported in Refs^3,4^, driven by the increasing complexity and decentralization of power grids. Researchers have been focusing on developing robust methods to address issues unique to distribution systems. Challenges and emerging trends in early fault detection include the integration of smart grid technologies, the use of big data analytics, and the development of hybrid detection approaches, as outlined in Ref^5^. A typical challenge with detection methods using meters at a single location is determining appropriate detection thresholds or creating representative datasets for machine learning-based approaches^6^. In practice, fault section identification (FSI) is hindered by the malfunction of monitoring devices, complicating the process^7^. The integration of renewable energy sources and active loads demands advanced monitoring infrastructure like smart meters and micro-phasor measurement units (µPMUs) to ensure power quality and system reliability while managing bidirectional energy flows in modern smart grids^8^. These advancements underline the critical role of innovative approaches in ensuring reliable and efficient fault detection in modern distribution networks. Hasan et al.^9^ provided an overview of the causes of power interruptions and the documented energy-management techniques. Electrical networks can experience various types of faults, as reported by William^10^. and Abdelhay et al.^11^, distribution system faults and failures are responsible for 94% of all power outages. 70% of blackouts have been caused due to relay failure, as documented by Arun et al.^12^ The distribution systems exhibit four primary fault types: Line-to-ground fault (L-GF), Line-to-line fault (LL-F), Line-to-Line-to-Ground Fault (LL-GF) and 3Line-to-Ground Fault (3 L-GF). L-GF occurs when one phase conductor makes contact with the ground, typically due to wind, animal interference, or a fallen line, such fault constitutes 70% of faults in distribution systems, and LL-F, caused by high winds, represents 15% of faults, also, LL-GF involves two phases and contributes to 10% of faults, as documented by Sophi et al.^13^ 3 L-GF, arising from equipment failure or tower collapse, is relatively uncommon, occurring at 5%, yet it poses significant risks due to the high fault current involved. Therefore, it’s essential to detect, classify and locate faults in the distribution system by using advanced protection relays. Several studies propose a signal processing technique-based protection strategy that enhances relay-tripping behavior.

To extract data from voltage or current signals, digital signal processing (DSP) techniques are essential. Furthermore, the ability to separate the transient components from the captured waveforms and process them to quickly identify the occurrence of fault transients is the fundamental principle behind these techniques. Because real-world signals frequently exhibit non-stationarity, processing in the time- or frequency-domains alone is insufficient because of how their spectral characteristics change over time. To improve signal analysis, this is addressed by both time-frequency approaches^14^. Fourier transform is the most common frequency domain technique, but it has two limitations as reported by Karlton et al.^15^ Wavelet technique is one of the most often used time-frequency methods that goes beyond the Fourier transform limitations. During the 20th century, researches on wavelet techniques (transforms) progressed gradually until the emergence of the continuous wavelet transform (CWT) in the 1980s, after which its advancement significantly accelerated. Moreover, the discovery of the discrete wavelet transform (DWT) and its inverse counterpart (IDWT) in the 1990s has enabled the compression and filtering of the faulted signals. The Discrete Wavelet Transform (DWT) is a wavelet technique commonly used in Digital Signal Processing (DSP)^16^. It is known for its effectiveness and adaptability, offering a perfect time-frequency localization for analyzing transient features during various fault types. This enables the construction of high-frequency and low-frequency components within a specified frequency band. The characteristics, benefits, drawbacks, and uses of wavelets have been shown in Tiantian, et al.^17^,and E. A. Frimpong, et al.^18^. Figure 1 provides a summary of wavelet classification, its applications, and associated challenges^19^. When operating with DWT, choosing the right decomposition level, mother wavelet, and combining it with machine learning are challenging tasks that must be done carefully^19^. Furthermore, the mother wavelet selection greatly influences the way it may be used to capture and represent complicated signal features across a variety of domains. The kind of information to be recovered from the signal and its characteristics determine which mother wavelet is the best.

Initially, it is crucial to choose an appropriate level. Various methods exist for determining the suitable decomposition level in the DWT, such as identifying the frequency bands with the highest percentage of signal energy^20,21^. When choosing the mother wavelet to study wind turbine health, two factors were used: the root mean square error and the energy to Shannon entropy ratio^22^. Heba at al^23^ utilize a method for selecting the optimal mother wavelet, integrating wavelet packet transform with the energy Shannon entropy ratio, offering a framework for the selection of optimal mother wavelet in human activity recognition. While the wavelet selection criterion relies on maximizing the cross-correlation coefficient^24^. The Minimum Description Length (MDL) approach to choosing the best mother wavelet^25^. Employing fuzzy logic to optimize the selection of mother wavelets^26^.Shannon entropy is employed to determine the optimal decomposition level for assessing wind turbine health^22^. The availability of 13 known families of mother wavelets means that the selection process can influence the outcomes of wavelet analysis. A qualitative approach considers properties such as regularity, compact support, symmetry, vanishing moment and orthogonality^27^. Due to the complexity of analyzing non-stationary signals, recent methodologies incorporate quantitative approaches for mother wavelet classification. The choice of the best method can vary based on factors such as signal properties, noise characteristics, and the specific goals of system analysis. Standard deviation and median absolute deviation are used to select the optimal mother wavelet^28,29^. While standard deviation and mean absolute deviation are used^30 ,31^. On the other hand, other works^20,21,32–34^ have employed the energy of wavelet coefficients as the criterion for optimal mother wavelet selection. This paper^35^ investigates the performance of various mother wavelets in a fault type classification algorithm for power systems, finding that different wavelets, including Daubechies, Symlets, Biorthogonal, and Coiflets, significantly affect the accuracy of fault detection by analyzing high-frequency signal components via discrete wavelet transform, with the highest accuracies noted for specific wavelets under distinct conditions.

The Support Vector Machine (SVM) is a robust supervised learning algorithm that has proven to be highly effective in a variety of applications, including classification and regression tasks. The significance of the support vector machine (SVM) algorithm lies in its ability to efficiently handle high-dimensional data while maximizing the accuracy of the classification task. SVM’s robustness in complex datasets makes it indispensable in tasks ranging from text categorization to image recognition. Model training times, accuracy, prediction speed, and absolutely are critical factors in achieving optimal results in machine learning^35–39^. High accuracy ensures reliable predictions, while faster prediction speeds enable real-time applications. Additionally, shorter model training times enhance efficiency, allowing for rapid iteration and deployment of machine learning solutions. Balancing these factors is key to achieving perfect results in various applications.

This investigation suggests valuable perspectives on the selection of wavelet levels and the choice of the suitable mother wavelet for using wavelet techniques in analyzing fault signals to detect the different fault cases. This involves combining wavelet analysis with a machine learning tool called SVM. The first step is to select a suitable decomposition level, where the wavelet transform method is used to decompose the standard fault signal into different wavelet components across multiple scales (11) and frequency bands (12). The energy of the present signal within each frequency band is subsequently determined by summing up the wavelets within each scale. Analysis reveals that the predominant distribution of signal energies occurs within the frequency bands spanning from 0.0195 kHz to 0.0391 kHz, from 0.0391 kHz to 0.0781 kHz, and from 0.0781 kHz to 0.1563 kHz. The proportions of energy released within these frequency bands for phase A are approximately 14.53%, 54.48%, and 20.49% of the total energies, respectively. The histograms depicting the distribution of energy across frequency bands for fault current signals were generated using MATLAB’s wavelet transformation energy calculation method. A special focus on analyzing the energy percentages is presented within the low-frequency band (D9-A9) and the dominant frequency band (D7–D9), as well as their patterns of variation. D8 is the highest percentage of energy. For this reason, this parameter was used to detect the fault conditions in the distribution system. The maximum value of (D8) will be the input to SVM. The suitable mother wavelet and wavelet function will be selected through accuracy, prediction speed and training time of Fine Gaussian SVM. The main contributions of the paper are listed as follows:

- The paper conducts a comprehensive analysis of the IEEE-16 bus system using *DWT* for improved fault detection accuracy.
- The study systematically selects the D8 wavelet decomposition level based on energy concentration as a novel criterion for enhancing accuracy and detection sensitivity in power distribution networks.
- A strong comparative evaluation of 53 wavelet functions identifies Sym3 as the most efficient for fault detection with SVM classifiers, enhancing detection accuracy and computational efficiency.
- The proposed method achieves 100% fault detection accuracy across symmetrical/asymmetrical faults, and various normal transient conditions such as load/capacitor/DG switching ensuring reliable detection/discrimination under various fault conditions, fault resistances and fault inception angles.
- A 9 MW Wind Farm is connected to Busbar 10, and multiple fault conditions are modeled to evaluate the system’s performance with 100% accuracy.
- Our study evaluates 25 different machine learning classifiers, providing a comprehensive comparison that goes beyond traditional approaches in fault detection research, and the analysis conducts that the Fine Gaussian SVM with a 5-fold cross-validation has the best validation and test accuracy, at 100%, and the quickest training time of 0.67957 s.
- The proposed method Provides practical guidelines for wavelet parameter selection confirming robustness in diverse operational scenarios.
- The study Introduces a reproducible, systematic approach to optimizing wavelet selection and SVM parameters, supporting improved fault detection outcomes in distribution networks.
- The approach is computationally efficient, suitable for real-time fault detection, avoiding reliance on resource-heavy techniques like deep machine learning, in addition to accuracy, our evaluation includes prediction speed and training time, offering a holistic perspective on classifier performance for real-time applications.

The rest of the manuscript is organized as follows: The “Discrete wavelet transform (DWT)” section provides an overview of the Discrete Wavelet Transform (DWT). The “System model description” section clarifies the behavior assessment for IEEE-16 bus tested system under different conditions, which is conducted using MATLAB/SIMULINK. The “Level selection” section shows wavelet level selection method. The “Mother wavelet selection” section present a technique for mother and function wavelet selection. The “Results” is dedicated to the results drawn. Finally, the paper concludes in “Conclusion” section, is dedicated to the conclusion of the proposed method.

## Discrete wavelet transform (DWT)

Mathematical analysis techniques known as wavelet transformations are employed to analyze data with features across different scales. These features can include transients, trends with gradual shifts, or frequencies that fluctuate over time. Wavelet transformations were primarily developed to address the limitations of the Fourier transform.

**Fig. 1:**
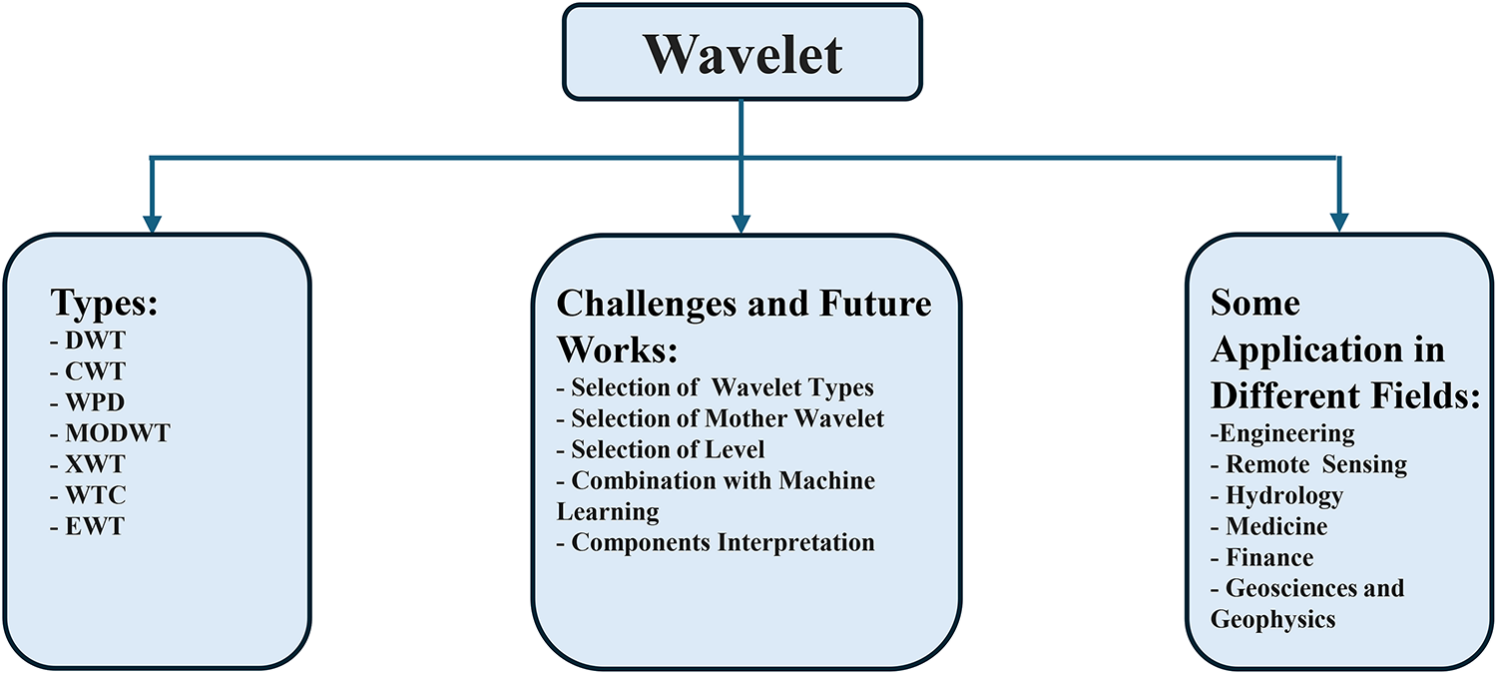
Types, challenges and application of wavelet.

The main objective of wavelet transformations is to analyze nonstationary signals, which are signals that exhibit a time-varying frequency response. A wavelet refers to a waveform with a finite duration and an average value of zero. The first step in the wavelet analysis procedure involves adopting a wavelet prototype function, also known as an analyzing wavelet or mother wavelet. This prototype function serves as a template for the wavelet analysis. By translating and scaling the mother wavelet, it is possible to represent any signal. The wavelet multiresolution analysis technique is particularly effective in analyzing travelling wave signals. DWT signal analysis allows for the selection of multiple scales (m) and positions (k) for combining wavelets, thereby enabling the formation of a signal at a specific scale of interest. By combining the results of the wavelet decomposition at all scales, the original signal can be perfectly reconstructed. This allows for the formation of a signal at a particular scale of interest. $\:f$($\:k$) represents the original signal or function that is subjected to the wavelet transform. In the context of this study, it corresponds to the transient fault current signal in the power distribution network, which is analyzed for fault detection. In multilevel wavelet analysis, the scale is successively halved at each level, resulting in a dyadic wavelet transform as described by Eq. (1)^35^.

1 $$DWT\left( {m,n} \right) = \frac{1}{{\sqrt {2^{m} } }}\mathop \sum \limits_{k} f\left( k \right)\psi [\frac{{n - k2^{m} }}{{2^{m} }}]~$$

Where: m, n, k = Integer values; $\:\psi\:=Mother\:wavelet$; n = number of data points; m = Scaling; k = Shifting.

**Fig. 2:**
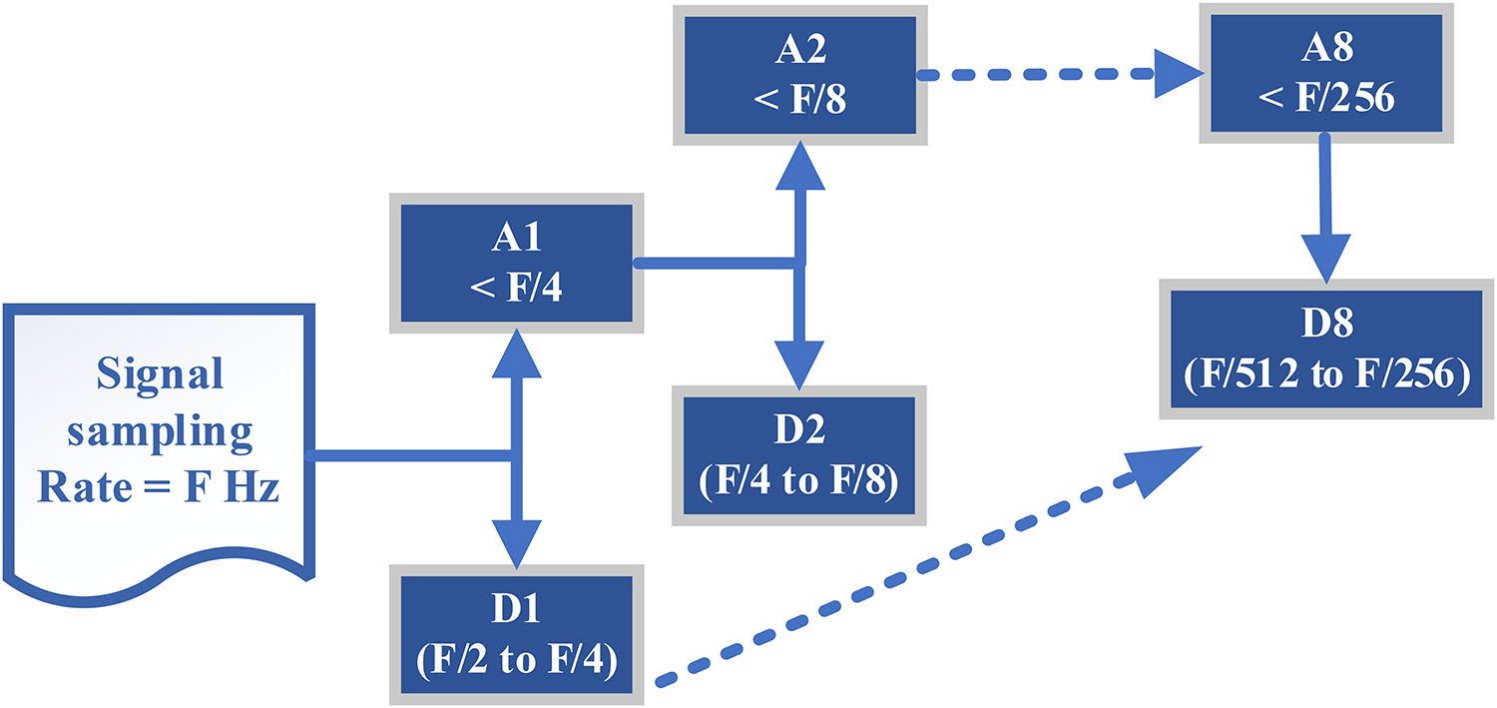
Frequency bands of each decomposition levels in DWT.

As shown in Fig. 2 in DWT, a signal is successively decomposed into approximation and detail coefficients through a series of high-pass and low-pass filtering operations, often referred to as the analysis filter bank. The signal’s finer characteristics are captured by the detail coefficients, while the coarser details are displayed by the approximation coefficients. By iteratively applying this decomposition to the approximation coefficients, an analysis with multiresolution may be conducted. The frequency ranges isolated by the (DWT) at each level adhere to the Mallat algorithm and Nyquist’s principle. According to the Nyquist theorem, asserting that the highest accurately representable frequency is less than half of the sampling rate, the maximum frequency of the original signal $\:f$($\:k$) sampled at F Hz is F/2 Hz^40^.

### Selecting discrete wavelet transform (DWT)

The choice of the DWT in our study was primarily driven by its ability to provide both time and frequency localization, making it particularly effective for analyzing non-stationary and transient signals, such as those generated by faults in power distribution networks. Fault signals are characterized by sudden changes in waveform patterns, and the Discrete wavelet transform is uniquely suited to capture these discontinuities with high precision. Many alternative signal processing techniques, such as the fast fourier transform (FFT)^41^ and Hilbert-Huang transform (HHT)^41,42^, have been widely used for signal decomposition. However, they are often limited in capturing transient or localized events due to their reliance on global frequency domain analysis (as in FFT) or computational complexity (as in HHT), and more recently, Deep Machine Learning (DML) approaches^43–46^. The wavelet transform, on the other hand, efficiently decomposes signals into multi-resolution components, making it highly effective for both fault detection and localization. To highlight key performance metrics Table 1 across these techniques, including their time-frequency resolution, computational efficiency, adaptability to non-stationary signals, noise robustness, and real-time applicability. This comparison underscores why DWT is often chosen for fault detection in power systems, especially where real-time processing and transient signal detection are required.

**Table 1:** Comparison between DWT and other signal processing techniques.

Comparison criteria	DWT	FT	STFT	HHT	DML methods	References (2022–2024)
Time-frequency resolution	High (adaptive, multiresolution)	Only frequency	Limited (fixed window size)	High (adaptive, non-linear)	High (depending on the model architecture)	R^41^ & R^42^
Non-stationary signal handling	Excellent for transients	Poor	Moderate	Excellent	Good (if trained on non-stationary data)	R^43^
Computational efficiency	Efficient for real-time	Efficient but lacks time information	Moderate (real-time possible)	Computationally expensive	Computationally expensive (training)	R^41^ & R^42^
Data requirements	Low (signal-driven)	Low	Low	Moderate (requires preprocessing)	High (requires large datasets)	R^43^
Interpretability	High (clear decomposition)	High (but no time localization)	Moderate	Moderate	Low (black-box nature)	R^44^& R^45^
Real-time applicability	Excellent for real-time	Not suitable for real-time	Moderate	Not suitable for real-time	Limited (depends on optimization)	R^46^
Noise robustness	High (denoising capabilities)	Moderate	Moderate	Low	Moderate to high (depends on preprocessing)	R^44^
Adaptability to fault types	High (captures different faults)	Low (no time information)	Moderate	High (adapts well to dynamic signals)	High (High (if trained on multiple fault types))	R^46^

*DWT* discrete wavelet transform,* FT  * fourier transform,* STFT*  short-time fourier transform,* HHT* Hilbert-Huang transform, *DML* deep machine learning.

### Wavelet-ML techniques overview

The combination of wavelet transform (WT) with support vector machines (SVM) has been widely used in the past and is considered a well-established approach. Our goal in utilizing this combination was to build upon its proven efficacy for fault detection, while leveraging the strengths of DWT in capturing transient signals and the robustness of SVM in classification tasks. While SVM is known for its strong theoretical foundations, offer better scalability and efficiency, especially when dealing with large datasets or real-time applications. This makes them more suitable for modern fault detection systems, where both accuracy and speed are critical, while recognizing the evolving landscape of fault detection methodologies in the literature^47–57^. Table 2 provide a critical evaluation of these approaches to the wavelet-SVM combination in terms of accuracy, computational complexity, and real-time feasibility.

**Table 2:** Summary of related works-based wavelet analysis with machine learning techniques.

Ref no.	Year	Feature extraction combinations	System typology	Rated voltage simulation via	Fault type	Accuracy (%)	Training time (S)
R^47^	2021	WPT + SVM	Eskom- power line	132 kV/MATLAB	LIFs	98.85	Nr
R^48^	2021	DWT + SVM	IEEE-33 bus	24.90 kV/DIgSILENT	LIFs	99.03	Nr
R^49^	2022	DWT + SVM	IEEE-33 bus	12.66 kV/MATLAB	LIFs	100	12.246
R^50^	2022	DWT + SVM	IEEE-33 bus	12.66 kV/MATLAB	LIFs	87.10	0.400
R^51^	2023	CNN + SVM	IEEE-123 bus	4.16 kV/MATLAB	LIFs	98.36	Nr
R^52^	2023	DWT + SVM	IEEE-9 bus	MATLAB/SIMULINK	LIFs	99.30	0.910
R^53^	2024	DWT + DNN	11-kV, Two-area cluster	MATLAB/SIMULINK	LIFs	100	Nr
R^54^	2024	DWT + RBFNN	IEEE-5 bus	MATLAB/SIMULINK	LIFs	100	Nr
R^55^	2024	RL + CNN (**DML**)	IEEE-33 bus	12.66 kV/MATLAB	HIFs	98.69	0.03261
R^56^	2024	ST + CNN (**DML**)	IEEE-13 bus	4.16 kV/MATLAB	LIFs + HIFs	99.73	Nr
R^57^	2024	DWT + DTE and ANN	Genuine isolated MV distribution	35 kV/MATLAB	LIFs	100	Nr

*CNN*   convolutional neural network,* DTE*  decision tree ensemble,* DNN*  deep neural network,* DWT*  discrete wavelet transform,* EML* ensembled machine learning,* DML* deep machine learning,* Nr* not reported,* RL* reinforcement learning,* RBFNN * radial basis function neural network,* WPT* wavelet packet transform,* ST * stockwell transform.

### DWT challenges and limitations

Computational complexity can pose a challenge in the application of the discrete wavelet transform (DWT), particularly for real-time fault detection and large-scale systems. The DWT performs multiresolution analysis by decomposing signals into various frequency bands, which, while highly effective for transient detection, increases computational load. As the number of decomposition levels increases to capture finer details, the processing time and memory requirements also grow. This can be a limitation for high-resolution data or real-time applications where timely analysis is crucial. Compared to other methods like the fast fourier transform (FFT), DWT is more computationally intensive, although FFT lacks the ability to capture time-localized events. Similarly, techniques like the Hilbert-Huang transform (HHT) are even more computationally expensive. Despite these challenges, DWT’s advantages in signal denoising, time-frequency localization, and fault detection accuracy make it a valuable tool. To mitigate complexity, optimized algorithms such as the fast wavelet transform (FWT) or hardware acceleration using Graphics processing unit (GPUs) or field programmable gate arrays (FPGAs) can be employed. With these optimizations, the DWT remains suitable for real-time fault detection despite its inherent computational demands^46^.

## System model description

**Fig. 3:**
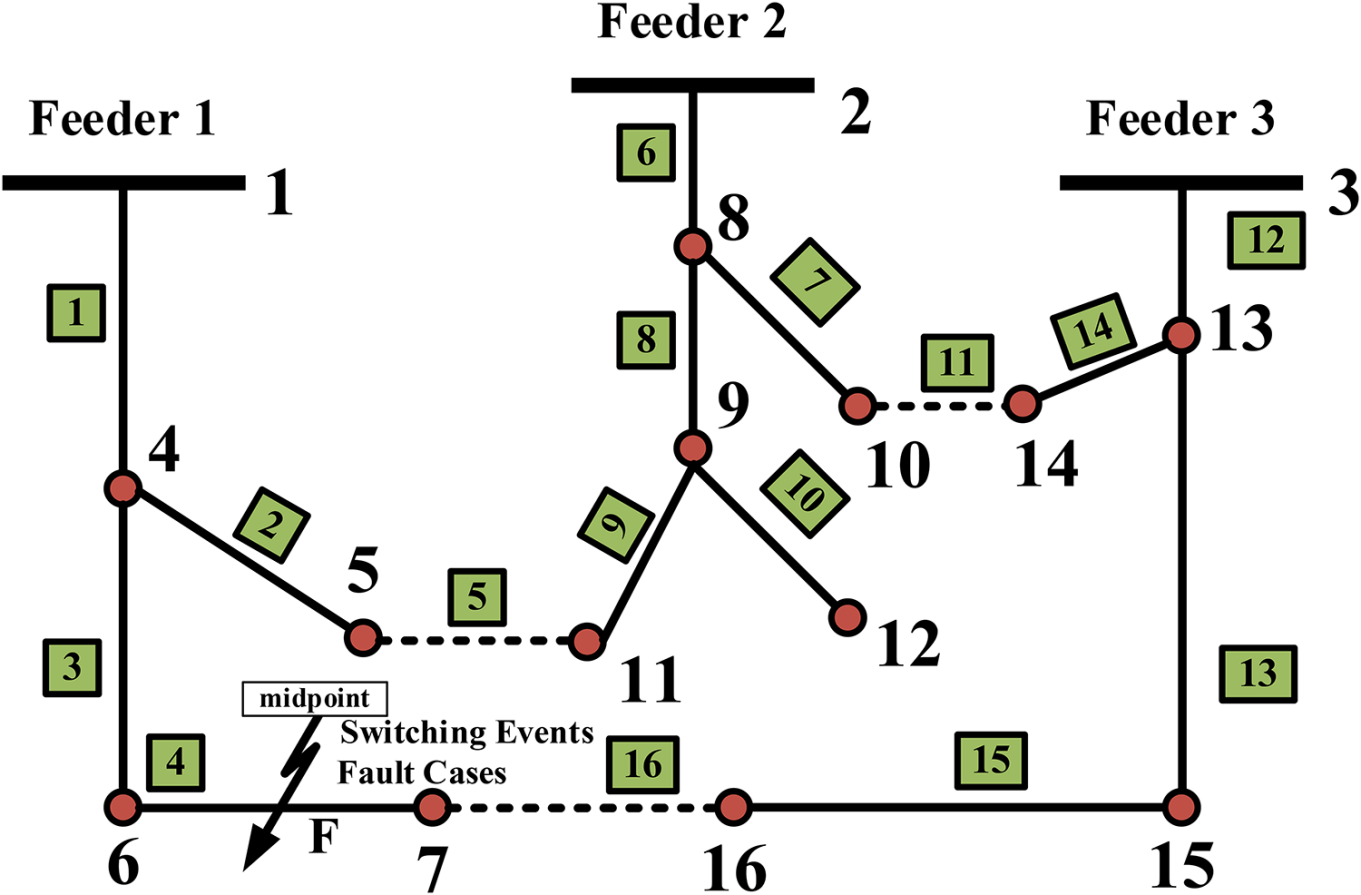
IEEE standard 16 bus tested system.

Figure 3 illustrates a 16-bus distribution system based on IEEE standard. The configuration comprises three feeders with a base power of 100 MVA and a base voltage of 23 kV. The overall system load is 28.7 MW and 17.3 MVAr, while the total reactive power injected across multiple loads amounts to 11.4 MVAr. The information pertaining to the line data and bus data of the IEEE 16-bus distribution system are presented in Table 3, as listed in^58^ MATLAB/Simulink environment is utilized to simulate the system model. Then, the fault signals are analyzed through MATLAB Wavelet Toolbox (MWT)to select suitable level. Finally, Using MATLAB Classifier Learner (MLC) to select the appropriate wavelet function for the classifier based on higher accuracy, faster prediction speed, and reduced training time.

### Case study of transient fault current signal

- Fault type: L-GF, LL-F, LL-GF, 3 L-F and 3 L-GF.
- Fault location: the fault occurs at the midpoint between lines 6–7 within the IEEE 16-bus system, and that it specifically involves a different fault types, as illustrated on (Fig. 3). IEEE standard 16 bus tested system. The fault is simulated at a distance of 50% along the line connecting. All the aforementioned types are examined at fault point and fault inception angles on the current waveform covering the protected line.
- The Fault resistance: 5, 10, 15, and 20 ohms.
- Fault inception angle: 0, 30, 45, 60, 90 and 120°.
- Switching cases: The performance of the proposed fault detection scheme based (wavelet transform and SVM classifiers) was evaluated under various normal switching conditions, including capacitor switching scenarios, Load switching scenarios, and DG (Diesel Generator) scenarios. However, their switching events can generate transients that might be misinterpreted as fault conditions by certain detection schemes. To validate the robustness and reliability of our proposed approach, we tested the scheme under controlled different switching events: -.

1. Capacitor switching of 3.7MVArs, that tested in two stage one at 40% of nominal value and the other case at 100% of nominal value to validate the performance of proposed scheme.
2. Load switching of 5 MW, that tested in two stage one at 20% of nominal load value and the other case at 40% of nominal load value.
3. DG switching (DG) at 10%, and 25% of the total rated power 28.70 MW.
4. A wind farm with a capacity of 9 MW is linked to Busbar 10, and diverse fault scenarios are analyzed to achieve perfect system performance evaluation.

**Table 3:** Bus data and line data.

Line	Section resistance (P.U)	Section reactance (P.U)	End bus load (MW)	End bus load (MVAR)	End bus capacitor (MVAR)
1–4	0.075	0.10	2	1.6	–
4–5	0.08	0.11	3	1.5	1.1
4–6	0.09	0.18	2	0.8	1.2
6–7	0.04	0.04	1.5	1.2	–
2–8	0.11	0.11	4	2.7	–
8–9	0.08	0.11	5	3	1.2
8–10	0.11	0.11	1	0.9	–
9–11	0.11	0.11	0.6	0.1	0.6
9–12	0.08	0.11	4.5	2	3.7
3–13	0.11	0.11	1	0.9	–
13–14	0.09	0.12	1	0.7	1.8
13–15	0.08	0.11	1	0.9	–
15–16	0.04	0.04	2.1	1	1.8
5–1	0.04	0.04	–	–	–
10–14	0.04	0.04	–	–	–
7–16	0.09	0.12	–	–	–

## Level selection

**Fig. 4:**
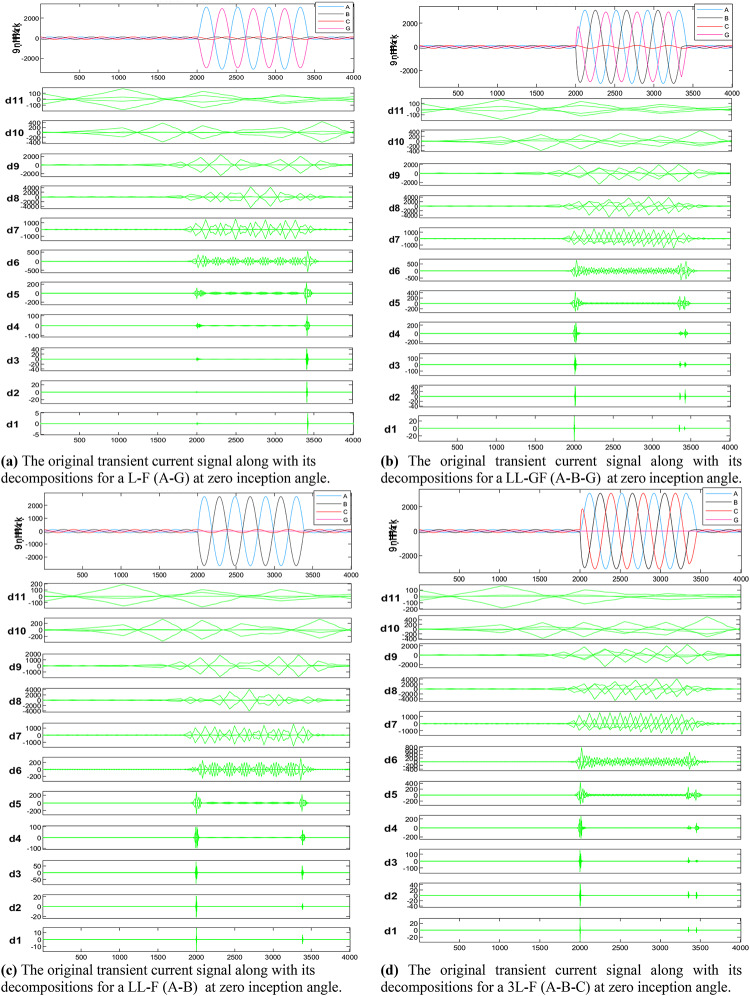

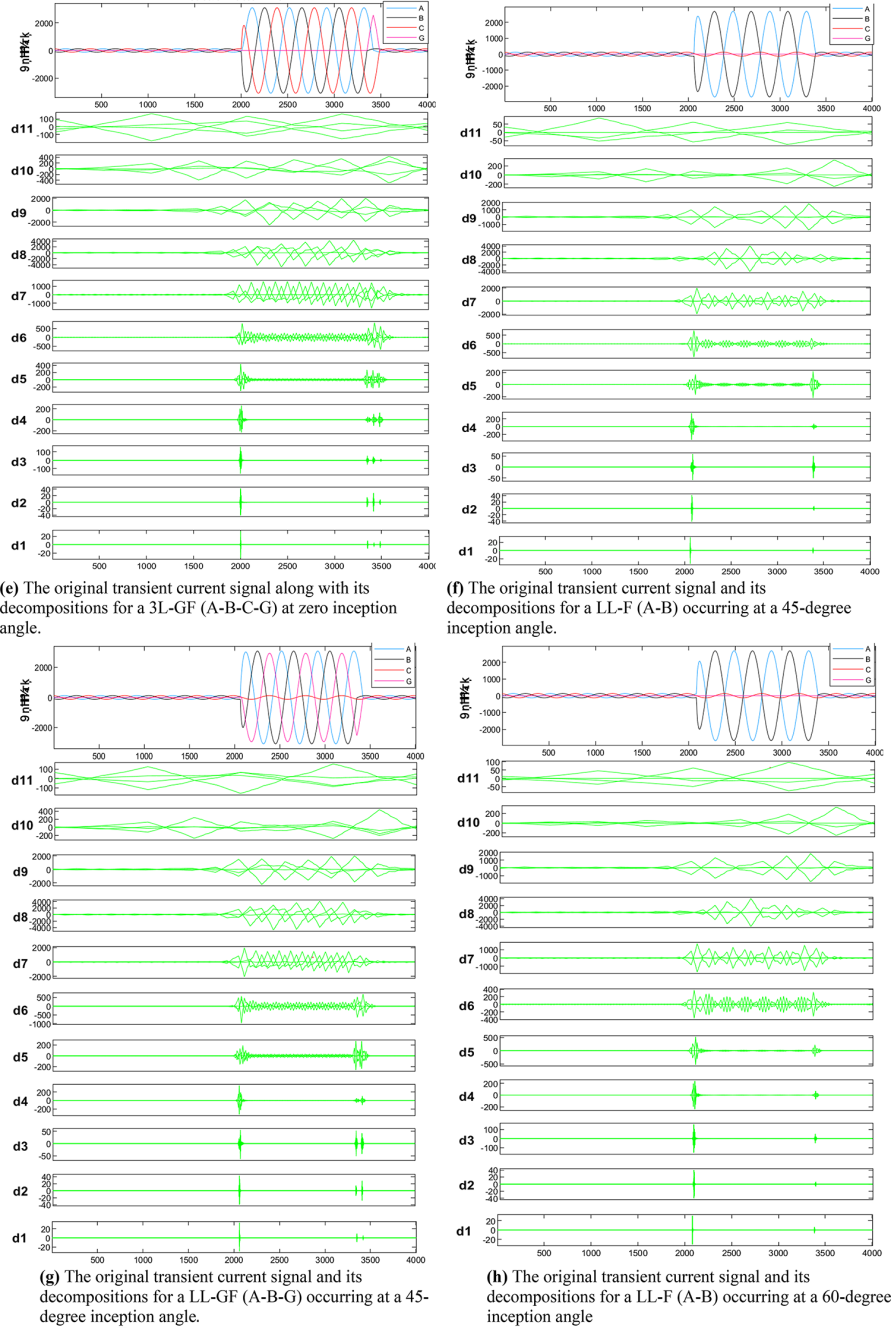

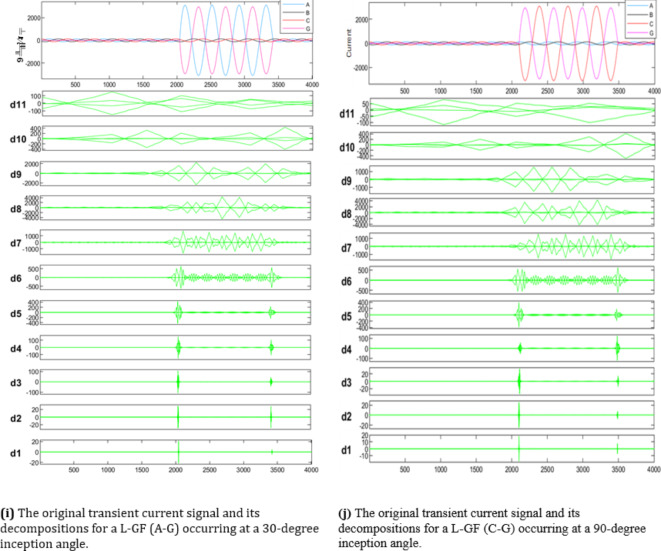
The original transient current signal and its decompositions for different faults conditions.

The MWT is used to analyze the current signal in order to select an appropriate wavelet level. As illustrated in Table 2 the lowest frequency band corresponds to the approximation scale (A11), while moving from (D11) to (D1) signifies an increase in frequency when transitioning from the approximate coefficient scale to the detail coefficient. Figure 4 shows the original transient current signal alongside its decompositions for various fault types at the midpoint of line 6–7. These fault types include L-GF, LL-F, LL-GF, 3 L-F, and 3 L-GF, all characterized by different inception angles (0, 30, 45, 60, 90 and 120°) and a fault resistance of 5, 10, 15, and 20 ohms. Analysis reveals that most energy is concentrated within three scales: detail D9 (b4), detail D8 (b5), and detail D7 (b6). These scales, encompassing 89.5% of the total signal energy, are indicative of distinct material damage mechanisms owing to their varied frequency bands. The sampling frequency is 20 kHz subsequently, frequencies ranging from 0 to 10 kHz are divided into 12 bands that are evenly spaced, in addition the transient current energy within each band, along with its proportion to the phase A transient current energy is computed, as listed in (Table 4). Results indicate that, in the recorded transient current signal, the majority of energy is contained within frequencies below 312.5 Hz, with minimal energy present beyond 625 Hz. Specifically, for the analyzed transient current signals, energy distribution is primarily observed within the bands spanning 0.01953–0.03906 kHz (b4), 0.03906–0.07812 kHz (b5), and 0.07812–0.15625 kHz (b6), with the (b5) frequency band emerging as dominant among the three, then the suitable mother wavelet will be detected at level 8 (D8).

## Mother wavelet selection

**Fig. 5:**
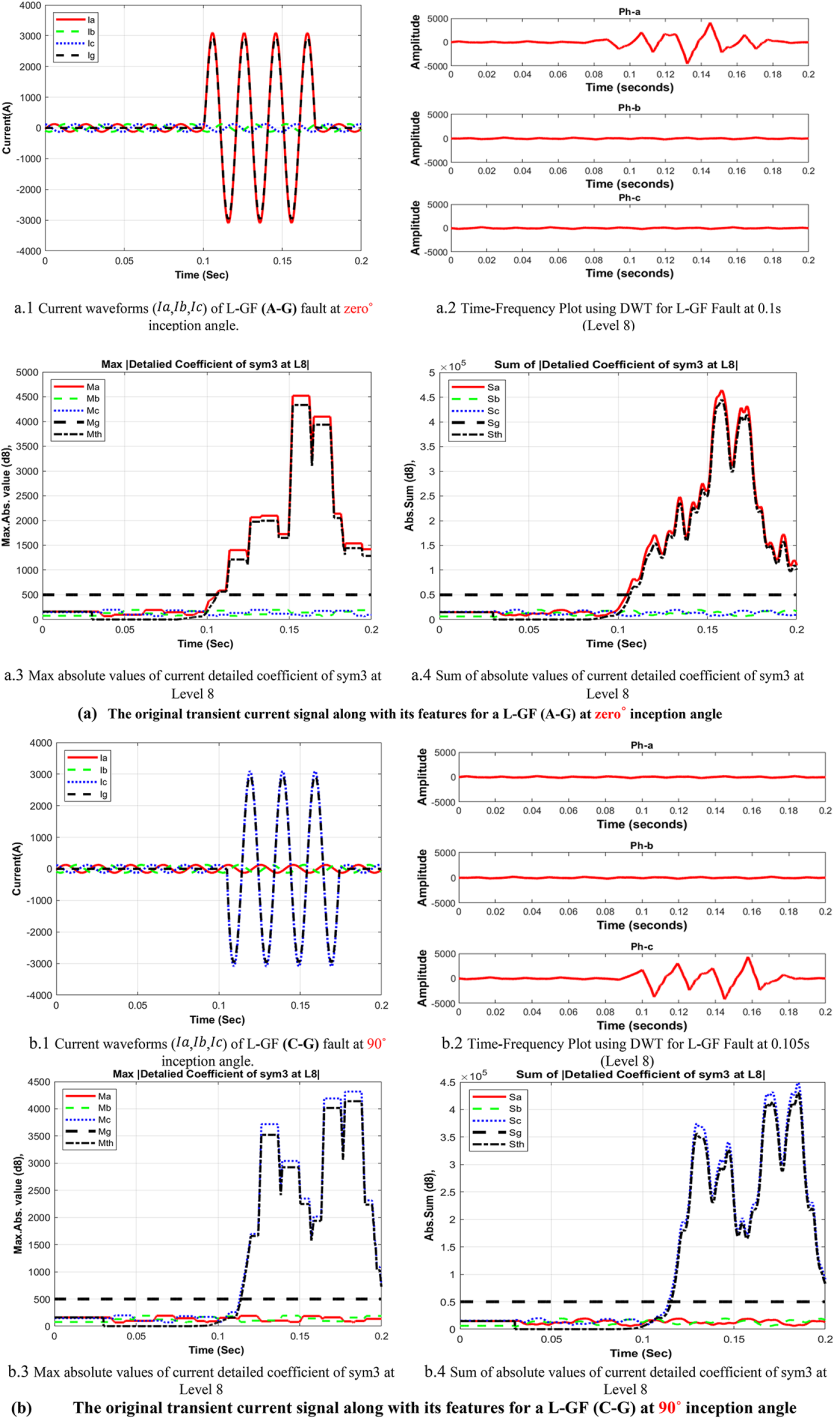

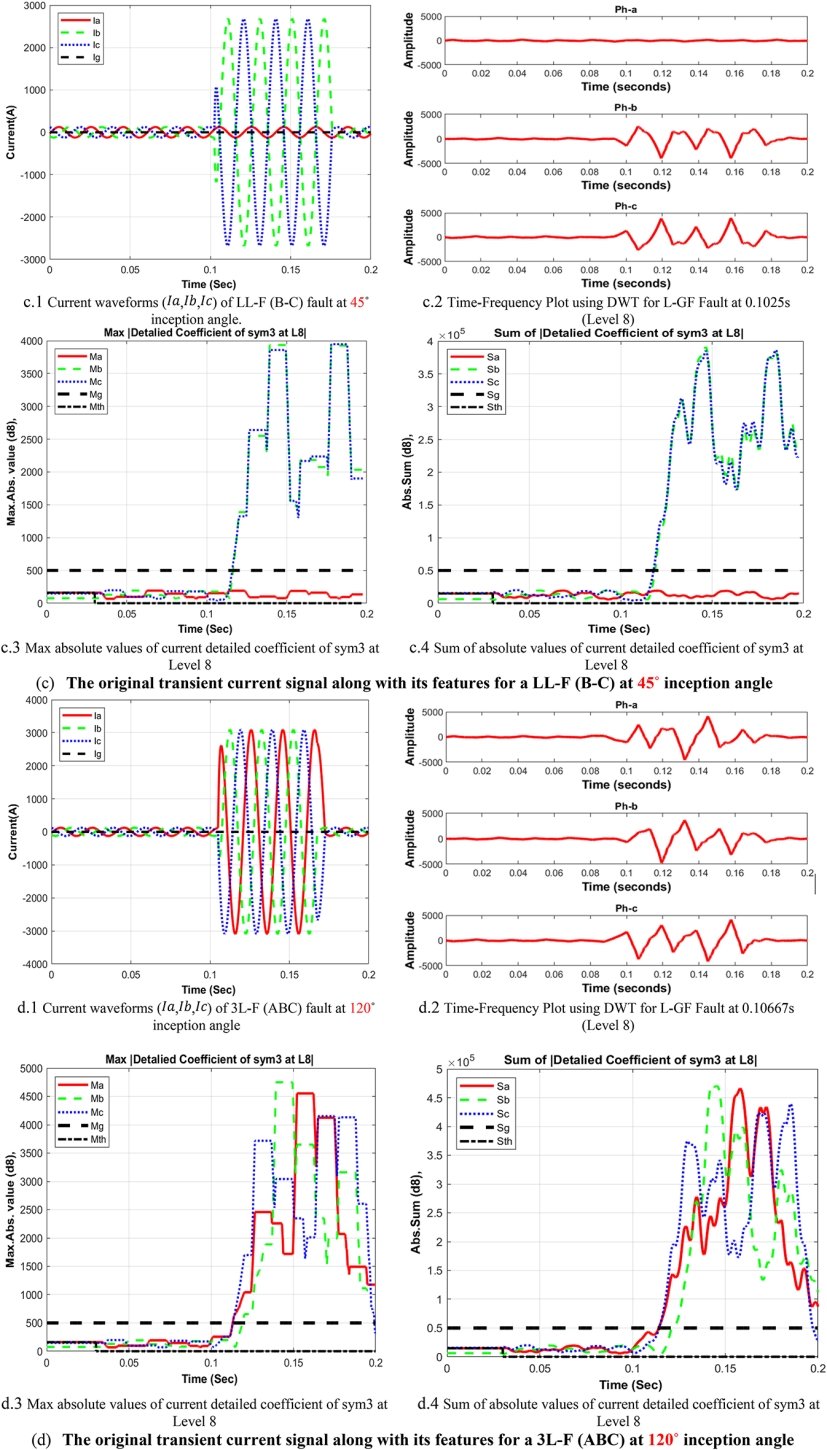
Measured current waveforms, Time-Frequency Plot using DWT, calculated Max absolute values of current detailed coefficient of sym3 at level 8 *(Ma*,* Mb*,* Mc*,* Mg)* and sum of absolute values of current detailed coefficient of sym3 at level 8 (*Sa*,* Sb*,* Sc*,* and Sg)* for different fault types, and different inception angles.

In the context of wavelet transform (WT), the significance of mother wavelets is paramount for analysis, with their diverse shapes tailored to specific applications. The critical challenge lies in selecting the most appropriate mother wavelet to ensure optimal results and a strong correlation between the signal and the chosen wavelet. The decomposed current detail coefficients of high-frequency component at estimated wavelet level 8 (D8) based on DWT, which applied in the fault detection algorithm using sym3 that shows a good correlation with the faulted signal. The main transient features of the suggested procedure will proceed as follows, the signal’s features for max absolute values of current detailed coefficient of sym3 at Level 8 (*Ma*,* Mb*,* Mc*,* and Mg*), and Sum of absolute values of current detailed coefficient of sym3 at Level 8 (*Sa*,* Sb*,* Sc*,* and Sg)*, are distributed across time that represent a high-frequency components (eight detail coefficient D8: is 0.0391∼0.0781 kHz), making it easier to understand the effect of different faults at different inception angles on the signal. The plots in Fig. 5, illustrate how the wavelet coefficients vary in response to different faults, showing how certain frequency components are more prominent during fault inception. The significant variation in the max absolute values of current detailed coefficient), and Sum of absolute values of current detailed coefficient of sym3 at Level 8 that indicate the variance in higher frequency bands during fault inception, this index is used to train and validate the machine learning classifier based on MATLAB Classifier Learner, to selecting the most appropriate mother wavelet. The study employs various wavelet families, including Haar, Daubechies, Coiflet, Symlets, Biorthogonal Reverse Biros Splines and Meyer wavelets. Key properties of wavelet families are outlined and discussed in^27^. Choosing the optimal mother wavelet is crucial in applications such as fault detection, as it greatly impacts the model’s classification accuracy, prediction speed, and training time.

### Key considerations for selecting a wavelet function

- Classification accuracy with Fine Gaussian SVM with cross validation = 5, and to ensure the reliability of our results, we implemented several measures to prevent overfitting. *First*, we applied 5-fold cross-validation during the training phase, where the dataset was split into four subsets, and the model was iteratively trained on four subsets while validated on the remaining one^59^, and 5-fold cross-validation provide a good balance between bias, variance, and computational efficiency, making them practical choices^60^. This ensured the model’s performance was robust across all data partitions. *Second*, the dataset was carefully balanced to include diverse fault scenarios, ensuring that all fault types, inception angles, and resistance values were equally represented. *Third*, the model was tested on completely unseen data, which was excluded during training, confirming its ability to generalize effectively. *Lastly*, we selected only the most relevant features extracted from the wavelet decomposition to reduce data dimensionality and prevent overfitting. These measures collectively validate the robustness and reliability of the reported results.
- The chosen wavelet must accurately capture the signal’s relevant features to achieve high classification accuracy.
- Using efficient wavelets reduces computational complexity, which in turn enhances accuracy, prediction speed and minimizes training time.

**Table 4:** Frequency range for each band.

Scale	Frequency range (kHz)	Frequency band no.	Percentage (%)
A11	0–0.00489	b1	0.92
D11	0.00489–0.00966	b2	0.49
D10	0.00978–0.01953	b3	1.27
D9	0.01953–0.03906	b4	14.53
D8	0.03906–0.07812	b5	54.48
D7	0.07812–0.15625	b6	20.49
D6	0.15625–0.31500	b7	5.77
D5	0.31250–0.62500	b8	1.54
D4	0.62500–1.2500	b9	0.39
D3	1.2500–2.5000	b10	0.1
D2	2.5000–5.0000	b11	0.02
D1	5.0000–10.000	b12	0.01
∑	–	–	100%

## Results

**Fig. 6:**
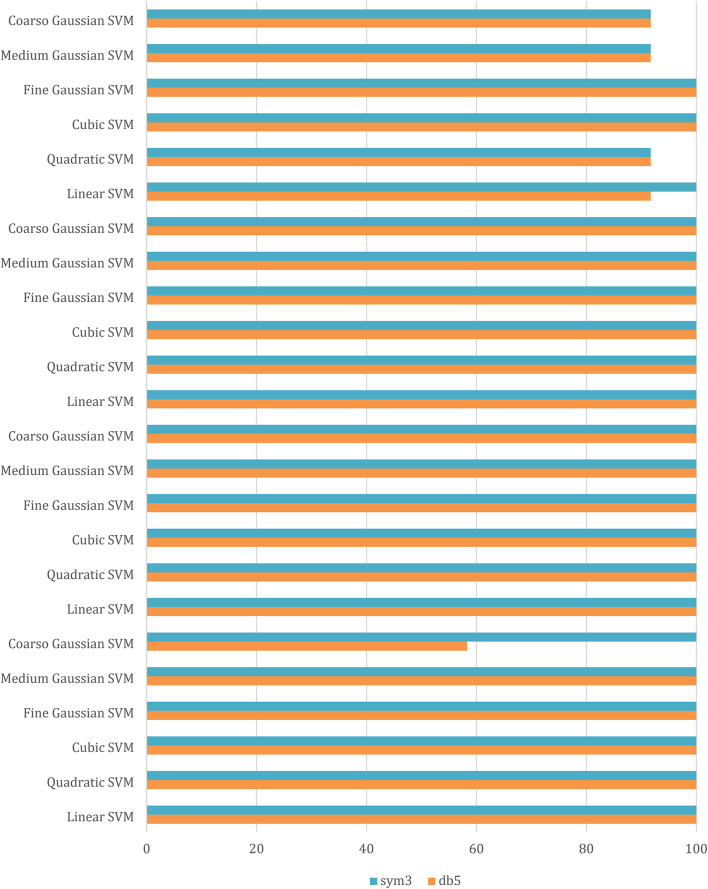
Accuracy (%) of SVM classifier for db5 & sym3 for phase A, B, C and ground at fixed resistance, fault location and fault inception angle.

**Fig. 7:**
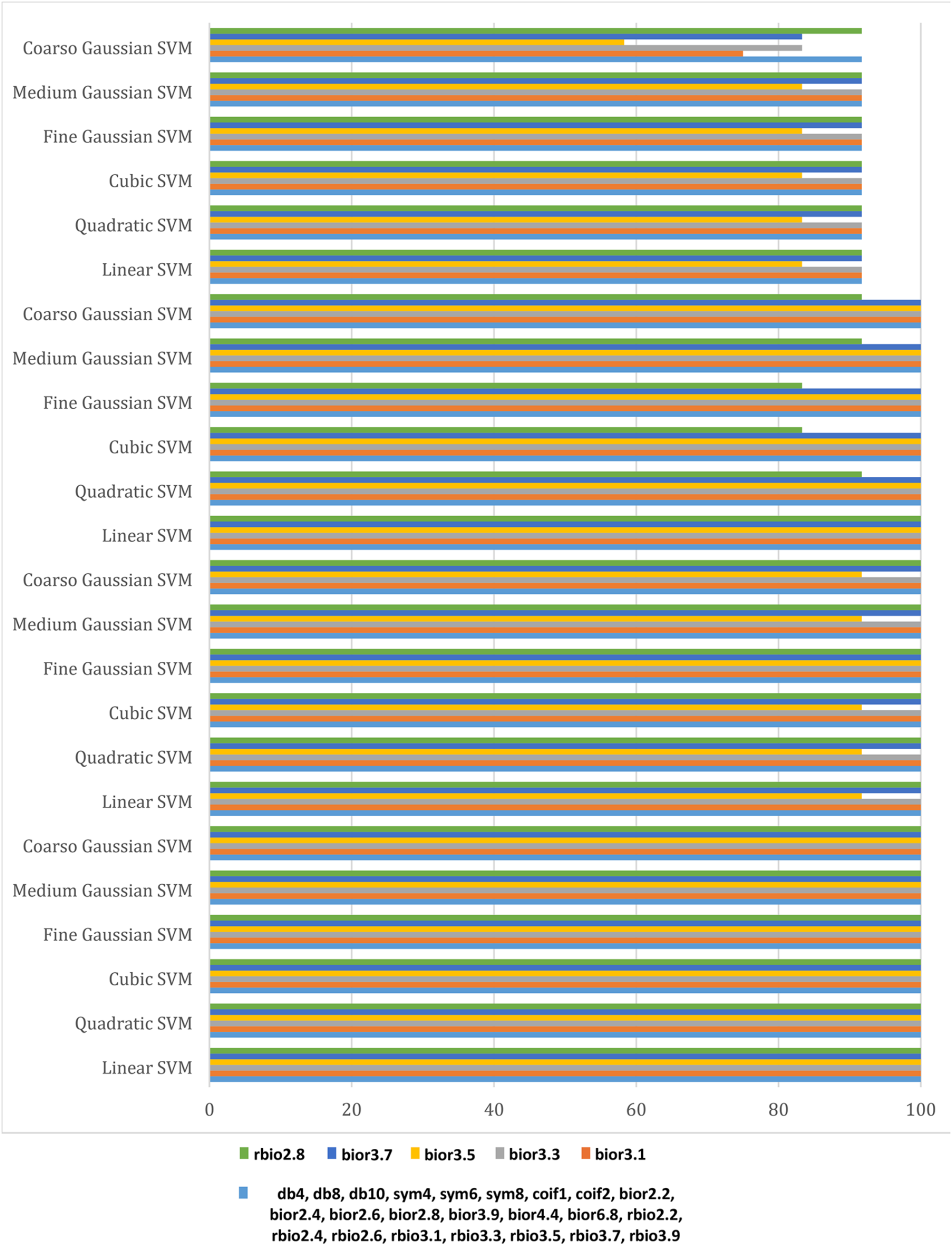
The accuracy (%) of the SVM classifier across 28 wavelet functions for phases A, B, C, and ground, taking into account fixed resistance, fault location, and fault inception angle.

**Fig. 8:**
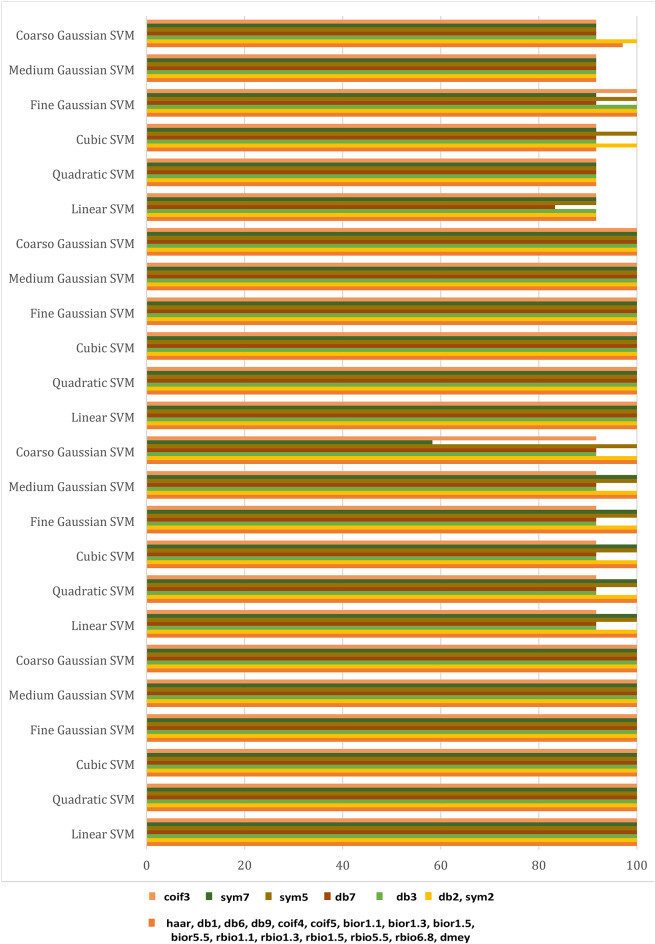
The accuracy (%) of the SVM classifier using 23 different wavelet functions for phases A, B, C, and ground, considering fixed resistance, fault location, and fault inception angle.

- As illustrated in Fig. 4, These figures demonstrate the transient fault current signals obtained from various fault scenarios and their corresponding wavelet transform decompositions at different levels, at each decomposition level (from the approximation to detail coefficients) the fault characteristics are captures to emphasizing how Level 8 decomposition provides optimal results in terms of distinguishing fault transients from normal conditions. The wavelet transform decomposes the current signals into different frequency bands at 11 levels. The guide to selecting suitable wavelet functions in DWT for Fault Detection methodology using the wavelet extracted signals index as classification criteria. For each 10 cycles at each level, the details coefficients are calculated and recorded. The classification criteria values are calculated for all mentioned levels and it is observed that classification features are more obvious on level 8, which correspond to frequency range from 0.0391 kHz to 0.0781 kHz.
- Figure 5: The figure demonstrates the measured current waveforms ($\:Ia$,$\:Ib$,$\:Ic$), Time-Frequency Plot using DWT, and the calculated corresponding transient features as a max absolute value of current detailed coefficient of sym3 at Level 8 (*Ma*,* Mb*,* Mc*,* and Mg*), and Sum of absolute values of current detailed coefficient of sym3 at Level 8 (*Sa*,* Sb*,* Sc*,* and Sg)* across time that represent a high-frequency components at different faults, and different inception angles on the signal. Also, the figure illustrates how the wavelet coefficients vary in response to different faults, showing how certain frequency components are more prominent during fault inception. The significant variation in the max absolute values of current detailed coefficient is used to train and validate the SVM classifiers with 53 wavelet functions, to selecting the most appropriate mother wavelet.
- Table 3: reveals that most energy is concentrated within three scales: detail D9 (b4), detail D8 (b5), and detail D7 (b6) in the recorded transient current signal, the majority of energy is contained within frequencies below 312.5 Hz, with minimal energy present beyond 625 Hz. Energy distribution is primarily observed within the bands spanning 0.01953–0.03906 kHz (b4), 0.03906–0.07812 kHz (b5), and 0.07812–0.15625 kHz (b6), with the (b5) frequency band emerging as dominant among the three, then the suitable level for a sym3 mother wavelet will be detected at level 8 (D8).
- Table 5: presents a summary of the classifier performance metrics, including Prediction speed, training time, and accuracy from Fine Gaussian SVM for 53 wavelet functions at one value of fault resistance, fault location, and fault inception angle for each classifier-wavelet combination. Daubechies 5 (db5) of Prediction speed 700 obs/sec, training time 0.63421 s, and accuracy 100% besides Symlet 3 (sym3) of Prediction speed 700 obs/sec, training time 0.67957 s, and accuracy 100% are identified as the best mother wavelets for training SVM classifiers with a 5-fold cross-validation setting. Daubechies 5 and Symlet 3 achieve perfect accuracy (100%) and discussing whether this could indicate potential overfitting. SVM classifier is strong theoretical foundations, offer better scalability and efficiency, especially when dealing with large datasets or real-time applications. This makes the SVM classifier more suitable for modern fault detection systems, where both accuracy and speed are critical.
- Table 6: For this comparison, we designed and trained a SVM model using the same dataset of wavelet-transformed fault signals. The SVM was tasked with learning patterns from the input wavelet coefficients to classify different fault types. We evaluated its performance on the same metrics as the traditional classifiers (accuracy, Training time, and Prediction speed). Preliminary results indicate that the SVM achieved comparable accuracy to the best-performing traditional classifiers (such as DT, KNN, Logistic Regression, Naive Bayes, Ensemble, etc.). Also, Table 5, presents a comparative analysis of training model types based on the accuracy of validations and tests at their respective training times. The Fine Gaussian SVM (SVM) with a 5-fold cross-validation has the best validation and test accuracy, at 100%, and the quickest training time of 0.67957 s.
- Figs. 6 and 7, and 8 demonstrate the performance of the SVM classifiers with 53 wavelet functions in selecting suitable wavelet function (mother wavelet) in DWT for fault detection methodology, that represent the comparative performance of the wavelet transform combined with SVM classifiers. These figures present a comparative analysis of training model types based on the accuracy of validations and tests at their respective training times. This assessment aids in the selection of the most optimal model. A five-fold cross-validation is used to protect against the occurrence of overfitting. As illustrated in Fig. 6, sym3 outperforms db5, achieving higher classification accuracy. Specifically, db5’s accuracy for phase A using the Coarse Gaussian SVM is reported at 58.8%.
- While db5 is effective, sym3 demonstrates superior performance in classification accuracy, especially for fault detection in current signals. Therefore, for this application, sym3 is recommended as the optimal mother wavelet. The critical step involves extracting the most significant feature from the sym3 mother wavelet at level8 (D8), which captures essential characteristics of the fault current.

**Table 5:** Prediction speed, training time, and accuracy from Fine Gaussian SVM for 53 wavelet functions at one value of fault resistance, fault location, and fault inception angle.

Wavelet function	Phase - G		
	Prediction speed (obs/sec)	Training time (sec)	Accuracy (%)
Haar family			
Haar	600	3.3741	100
Daubechies wavelet family			
db1	600	3.3741	100
db2	440	2.3954	100
db3	550	3.2833	100
db4	740	0.61119	91.7
db5	700	0.63421	100
db6	580	0.67559	100
db7	580	0.73023	91.7
db8	590	0.66996	91.7
db9	690	0.69557	100
db10	940	1.6074	91.7
Symlets wavelet family			
sym2	580	1.2705	100
sym3	700	0.67957	100
sym4	640	0.6515	91.7
sym5	600	1.6276	100
sym6	610	2.3446	91.7
sym7	660	2.2513	91.7
sym8	440	2.67	91.7
Coiflets wavelet family			
coif1	680	2.5993	91.7
coif2	660	2.4117	91.7
coif3	510	3.5726	100
coif4	550	2.3771	100
coif5	320	5.0333	100
Bior splines wavelet family			
bior1.1	520	3.667	100
bior1.3	540	2.5186	100
bior1.5	560	2.6072	100
bior2.2	670	2.6757	91.7
bior2.4	580	2.7752	91.7
bior2.6	350	3.925	91.7
bior2.8	590	2.7764	91.7
bior3.1	580	4.2407	91.7
bior3.3	660	2.7482	91.7
bior3.5	510	3.7175	83.3
bior3.7	450	4.6526	91.7
bior3.9	690	4.0694	91.7
bior4.4	440	4.0722	91.7
bior5.5	610	4.0082	100
bior6.8	660	3.5318	91.7
Reverse bior splines wavelet family			
rbio1.1	630	2.2263	100
rbio1.3	180	5.0493	100
rbio1.5	160	5.1161	100
rbio2.2	620	4.4052	91.7
rbio2.4	160	4.9882	91.7
rbio2.6	680	4.9001	91.7
rbio2.8	90	4.1408	91.7
rbio3.1	580	2.6892	91.7
rbio3.3	420	5.4213	91.7
rbio3.5	700	5.1709	91.7
rbio3.7	670	4.4109	91.7
rbio3.9	530	4.8647	91.7
rbio4.4	370	4.5904	91.7
rbio5.5	680	5.2904	100
rbio6.8	200	5.2157	100
Meyer wavelet family			
dmey	510	6.2002	100

### Challenges of the proposed technique

The proposed technique is robust and effective; however, several challenges and limitations are recognized, and ongoing efforts aim to address them. To check its validity and capability to solve these challenges, as:

1. Adaptive decomposition levels: While a fixed decomposition level (D8) was optimal for the IEEE-16 bus system, an adaptive approach could enhance fault detection accuracy across different network configurations.
2. Generalization across network types: The scheme’s applicability to other network types, such as transmission or microgrid systems, requires further investigation and adjustments.
3. Real-time implementation: Transitioning the method from simulation to real-time applications will require efficient algorithms and high-speed processing capabilities.

**Table 6:** Comparison between the SVM classifier and other machine learning methods for detection/classification faults.

Model no.	Model type	Preset	Accuracy (%)	Error (%)	Training time (Second)	Prediction speed (obs/second)
1	SVM	Fine gaussian	100	0.00	**0.67957**	**700**
		Quadratic SVM	91.7	8.30	0.97779	730
		Medium gaussian	91.7	8.30	3.4325	690
		Cubic SVM	91.7	8.30	2.8381	740
2	Decision tree (DT)	Fine tree	83.3	16.70	4.3861	780
		Medium tree	83.3	16.70	3.4032	740
		Coarsetree	83.3	16.70	2.8681	910
3	Nearest neighbour (KNN)	Fine KNN	91.7	8.30	3.1783	350
		Medium KNN	58.3	41.70	1.5588	340
		Coarse KNN	58.3	41.70	1.0834	330
		Weighted KNN	91.7	8.30	0.94285	340
4	Logistic regression	Binary GLM logistic regression	91.7	8.30	1.0498	630
		Efficient logistic regression	100	0.00	**2.52778**	820
5	Naive bayes	Kernel naïve bayes	100	0.00	**1.4785**	430
		Gaussian naïve bayes	100	0.00	**2.8329**	540
6	Ensemble	Boosted trees	58.3	41.70	3.3585	810
		Bagged tree	91.7	8.30	2.8146	90
		RUBoosted trees	66.7	33.33	2.8232	350
7	Kernel	SVM kernel	58.3	41.70	2.851	600
		Logistic Regression Kernel	58.3	41.70	2.0485	770
8	Discriminant analysis	Quadratic discriminant	100	0.00	**2.8629**	410
9	Efficient linear SVM	Efficient linear SVM	91.7	8.30	0.92646	750
10	Neural network	Narrow neural network	91.7	8.30	4.2862	750
		Bilayered neural network	91.7	8.30	1.9436	610
		Trilayered neural network	91.7	8.30	1.1095	640

## Conclusion

The paper proposes a technique for selecting the decomposition level and wavelet function in wavelet techniques using MWT, particularly DWT for fault signal analysis, to enhance power distribution network fault detection. The appropriate level is identified by detecting the highest energy percentage in detail coefficients, with the most energy found in scales D9 (b4), D8 (b5), and D7 (b6), and D8 having the highest concentration. The maximum value of D8 will be used as input to SVM classifiers. The optimal mother wavelet is chosen based on the training time, prediction speed, and accuracy of the SVM classifiers, with sym3 wavelets proving to be the most effective. Extensive simulation tests are carried out to investigate the performance of proposed technique. The proposed approach reliably differentiates normal switching operations from fault events, whether distributed generation (DG) is present or absent. This highlights the robustness of the wavelet transform in analyzing transient signals and the effectiveness of the SVM classifier in making precise classifications, even under challenging operating conditions.

## Electronic supplementary material

Below is the link to the electronic supplementary material.

Supplementary Material 1

## Data Availability

The data that support the findings of this study are available from the corresponding authors upon reasonable request.
