# Supplementary material for: A comprehensive guide to selecting suitable wavelet decomposition level and functions in discrete wavelet transform for fault detection in distribution networks
Source: Sci Rep. 2025 Jan 7;15:1160. doi: 10.1038/s41598-024-82025-2 (PMC11706944; doi:10.1038/s41598-024-82025-2)
Supplement: Supplementary file 1 — Supplementary Material 1 [file 41598_2024_82025_MOESM1_ESM.docx]

**Acronyms/Nomenclature**

| *A9, and A11* | Approximation coefficients |
| --- | --- |
| *b1 to b11* | Frequency bands |
| *CWT* | Continuous Wavelet Transform |
| *CNN* | Convolutional Neural Network |
| *D1 to D11* | Detail coefficients |
| *db5* | Daubechies mother wavelet with order 5 |
| *DNN* | Deep Neural Network |
| *DML* | Deep Machine Learning |
| *DT* | Decision Tree |
| *DTE* | Decision Tree Ensemble |
| *DWT* | Discrete Wavelet Transform |
| *DSP* | Digital Signal Processing |
| *EWT* | Empirical Wavelet Transform |
| *EML* | Ensembled Machine Learning |
| *FT* | Fourier Transform |
| *FFT* | Fast Fourier Transform |
| *FWT* | Fast Wavelet Transform |
| *FPGAs* | Field Programmable Gate Arrays |
| *GPU* | Graphics processing unit |
| *HHT* | Hilbert-Huang Transform |
| *KNN* | K-Nearest Neighbour |
| *IDWT* | Inverse Discrete Wavelet Transform |
| *L-GF* | Line-to-Ground Fault |
| *LL-F* | Line-to-Line Fault |
| *LL-GF* | Line-to-Line-to-Ground Fault |
| *3L-GF* | 3Line-to-Ground Fault |
| *MDL* | Minimum Description Length |
| *MLC* | MATLAB Classifier Learner |
| *MODWT* | Maximal Overlap Discrete Wavelet Transform |
| *MWT* | MATLAB Wavelet Toolbox |
| *m, and k* | Scaling, and shifting respectively |
| *Ma, Mb, Mc, and Mg* | Calculated Max absolute values of current detailed coefficient of sym3 at Level 8 |
| $n$ | Number of data points |
| *RBFNN* | Radial Basis Function Neural Network |
| *RL* | Reinforcement learning |
| *sym3* | Symlets mother wavelet with order 3 |
| *SVM* | Support Vector Machine |
| *ST* | Stockwell transform |
| *Sa, Sb, Sc, and Sg* | Sum of absolute values of current detailed coefficient of sym3 at Level 8 |
| *WTC* | Wavelet Transform Coherence |
| *WPT* | Wavelet Packet Transform |
| *f(k)* | Original signal of three-phase and ground current signals |
| *XWT* | Cross-wavelet transform |
| $\psi$ | Mother wavelet |
